# Recent advances in aptamer-based therapeutic strategies for targeting cancer stem cells

**DOI:** 10.1016/j.mtbio.2023.100605

**Published:** 2023-03-11

**Authors:** Biao Liu, Jiahao Liu, Xing Hu, Wei Xiang, Weibin Hou, Chao Li, Jinrong Wang, Kun Yao, Jin Tang, Zhi Long, Long Wang, Jianye Liu

**Affiliations:** a Department of Urology, The Third Xiangya Hospital of Central South University, No.138, Tongzipo Road, Changsha, 410013, Hunan, China; b Department of Hepatopancreatobiliary Surgery, The Third Xiangya Hospital of Central South University, No.138, Tongzipo Road, Changsha, 410013, Hunan, China

**Keywords:** Aptamer, Cancer stem cells, Drug delivery system, Drug targeting, Immunotherapy, Multifunction

## Abstract

Cancer stem cells (CSCs) are believed to be the main cause of chemotherapy resistance and tumor relapse. Various therapeutic strategies to eliminate CSCs have been developed recently. Aptamers, also called “chemical antibodies”, can specifically bind with their molecular targets through special tertiary structures. The advantages of aptamers, such as lower immunogenicity and smaller size, make them superior to conventional antibodies. Therefore, aptamers have been used widely as targeting ligands for CSC-targeted therapeutic strategies in different tumor types. To date, various therapeutic cargoes have been conjugated to aptamers to kill CSCs, such as chemotherapy drugs, small interfering RNAs, and microRNAs. Aptamer-based targeted therapies for CSCs have made great progress in recent years, especially the development of multifunctional aptamer-based therapeutic strategies. Besides, cell-systematic evolution of ligands by exponential enrichment has been applied to screen new aptamers that might have a higher binding ability for CSCs. In this review, we focus on recent advances and introduce some new modalities of aptamer-drug conjugates against CSCs. Some considerations of the advantages and limitations of different aptamer-based targeted therapies for CSCs are also discussed.

## Graphical abstract

Different modalities of aptamer based therapeutic strategies for cancer stem cells. Firstly, aptamers can inhibit the growth of CSCs by blocking the related pathways. By conjugating drugs and siRNAs to aptamer, ApDCs have been developed for chemotherapy and gene therapy. Dual-aptamer or the combination of aptamer and other kinds of targeting ligands have been designed for co-targeting CSCs and non-CSCs. For combination therapy, aptamers guide two therapeutic agents to target CSCs. photothermal transduction agents are conjugated to dual-aptamer for simultaneous elimination of CSCs and non-CSCs. At last, as nucleic acid drug, AS1411 aptamer can be delivered by peptides.

**Figure:**
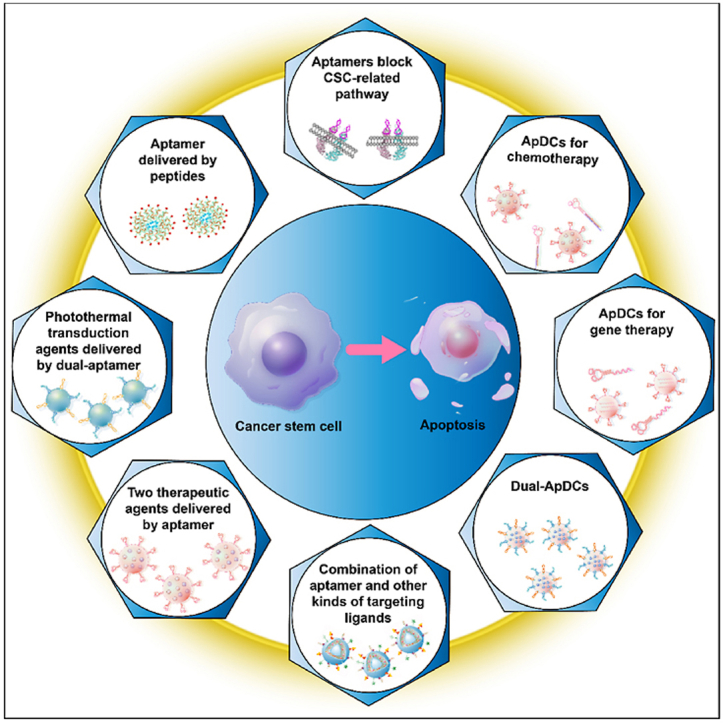

## 1 Introduction

Cancer stem cells (CSCs), a subset of cancer cells, occupy a small part in the mass of the tumor, but play an important role in tumor initiation and progression [[1], [2], [3]]. The main characteristics of CSCs are their self-renewal ability, tumor-generating potential, and the capacity to differentiate into all kinds of cancer cells, which are also used to distinguish CSCs from non-CSCs [4]. In recent decades, increasing evidence has revealed that the existence of CSCs might lead to tumor relapse and chemotherapy resistance, thus CSC-targeted therapeutic strategies have become a hot research topic [[5], [6], [7]]. However, CSCs are confirmed to dwell in the CSC niche, a strong barrier that hinders drug delivery, making it hard to eliminate all CSCs [8,9]. Moreover, phenotype reversal, the conversion between non-CSCs and CSCs, increases the difficulty of killing CSCs [10].

Therapeutic strategies aiming at targeting and eliminating CSCs, including the targeting of CSC biomarkers, the CSC niche, and CSC signaling pathways, have been developed in recent years [[11], [12], [13]]. Researchers in this field have tried to find the most efficient method to kill CSCs. Identifying and isolating CSCs from the mass of the tumor remains a challenge; however, the differences in the expression levels of some biomarkers between CSCs and non-CSCs provides potential solutions. For example, the expression of CD133, CD44, CD24, and epithelial cell adhesion molecule (EpCAM), and the activity of aldehyde dehydrogenase (A1DH), are commonly used to recognize CSCs [14,15]. By targeting these biomarkers, we can easily isolate CSCs from the whole tumor tissue [16,17].

Antibodies are usually considered an appropriate method to target cancer cells because they can specifically bind to the overexpressed biomarkers, and certain types of antibody-drug conjugates (ADCs) have been developed for cancer treatment [[18], [19], [20], [21], [22]]. Meanwhile, aptamers, known as “chemical antibodies”, have also been used as targeting ligands for drug delivery systems. Compared with conventional antibodies, aptamers are easier to modify with therapeutic components, such as drugs and small interfering RNAs (siRNAs). Besides, aptamers have better biocompatibility because of their low immunogenicity and higher permeability related to their small size [[23], [24], [25], [26]]. Moreover, molecular targets that are hard to generate antibody for can also be targeted by aptamers [27]. These advantages have led to the rapid development of aptamer-based therapeutics in recent years [28]. Aptamers against EpCAM, CD44, and CD133 have been used widely as targeting ligands to guide therapeutic agents to CSCs in different tumor types [[29], [30], [31]]. New aptamers are also being screened using the Cell-Systematic Evolution of Ligands by Exponential Enrichment (Cell-SELEX) method to find more applicable sequences [32,33].

To achieve a satisfactory anticancer effect, aptamers are often modified with therapeutic components. In addition to chemotherapy, CSC-targeted aptamer-based therapies have been developed for gene therapy, immunotherapy, and photothermal therapy (PTT) [34,35]. Considering that phenotype reversal between CSCs and non-CSCs increases the risk of tumor relapse, some researchers have developed dual-aptamer-drug conjugates (dual-ApDCs), in which one aptamer selectively binds to CSC biomarkers and the other one targets non-CSCs [36]. In addition, the combination of aptamers and other kinds of targeting ligands has been explored to co-target CSCs and non-CSCs [37].

Research in this area has made great progress in the last five years, especially the development multifunctional CSC-targeted ApDCs, and ongoing research brings the hope of overcoming drug resistance and tumor regrowth [38]. In this review, we summarize the different therapeutic strategies as cargoes to target and kill CSCs and discuss the different modalities of multifunctional CSC-targeted ApDCs. Finally, the perspectives and challenges of aptamer-based therapies to eliminate CSCs are discussed.

## 2 Aptamers as therapeutics for CSCs

**Fig. 1:**
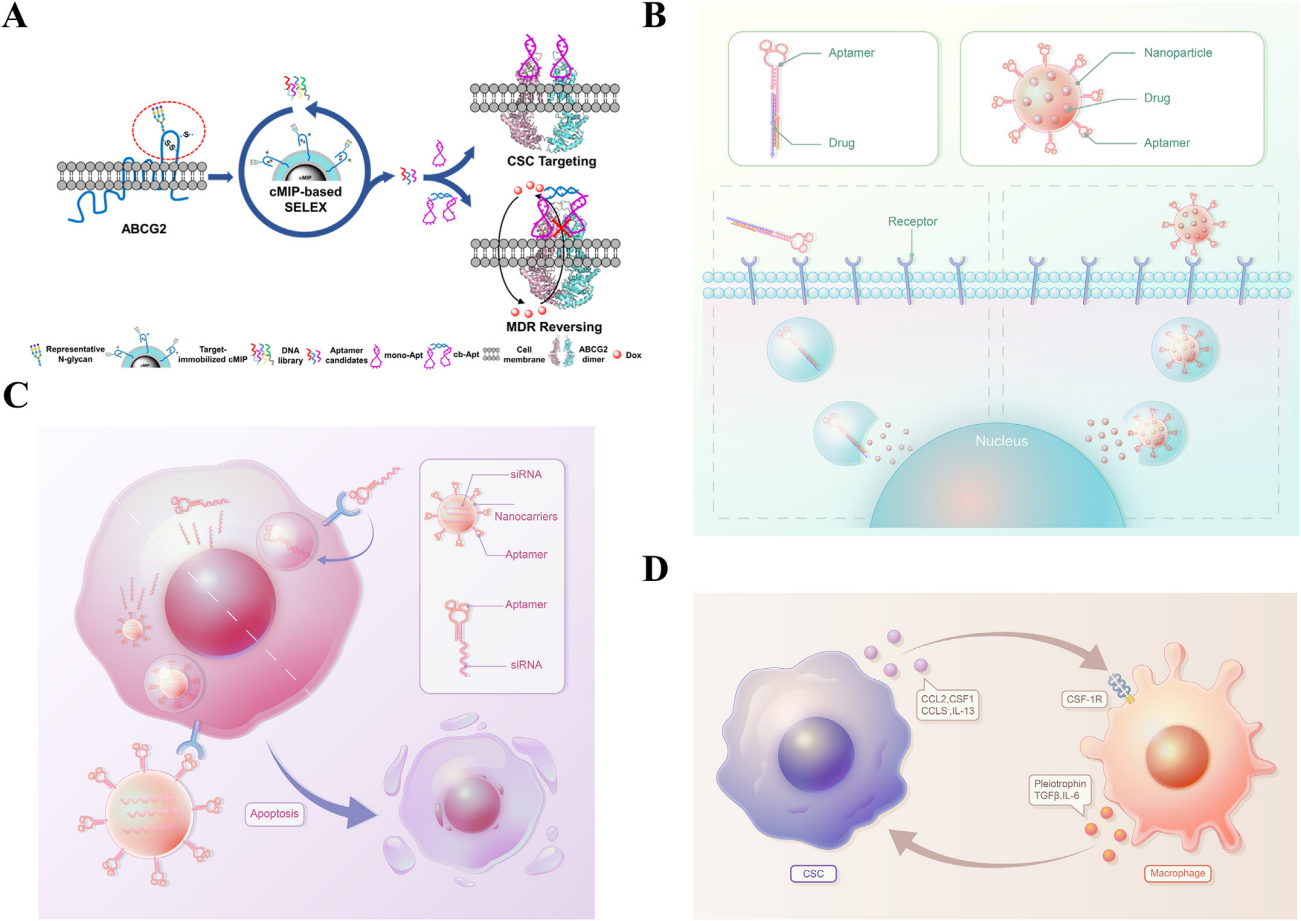
Single aptamer based CSC-targeted therapeutic strategies. (A) Aptamers reverse multidrug resistance. Aptamers bound to ABCG2 and blocked the ABCG2-mediated drug-pumping channel, thereby leading to the increased intracellular accumulation of drugs. Reprinted with the permission from Ref. [45]. **(B)** Aptamers serve as a targeting ligand or drug carrier to transport drug to CSCs. **(C)** A schematic representation of aptamer-modified nanocarrier for siRNA delivery for CSC-targeted therapy. **(D)** The interaction between CSCs and TAMs. CSCs recruit monocytes and macrophages via molecules including CCL2, CCL5, and CSF1, which bind to surface receptors, such as the CSF1 receptor (CSF1R). Macrophages, in turn, express factors including IL-6, pleiotrophin, and TGFβ to support CSCs.

Blocking protumoral signaling pathways by antibodies has demonstrated the prospects of suppressing tumor progression and metastasis [39,40]. Likewise, by modulating the related signaling pathways, aptamers against CSC-related biomarkers have shown promise in inhibiting the formation of CSCs and the migration of tumors (Fig. 1A) [[41], [42], [43]]. AP-9R is an example, which was developed using cell-SELEX to target lung CSCs. To screen DNA aptamers that specifically bind to lung CSCs, Wu et al. used lung CSCs, modeled by E-cadherin-silenced A549 ​cells, as target cells for cell-SELEX. After 15 rounds of selection, AP-9R was selected because of its high binding affinity for lung CSCs. Then, pull-down assays and mass spectrometry were performed to identify the target molecule of AP-9R, which suggested that stemness-related Annexin A2 is the protein target of AP-9R. *In vitro*, compared with the control aptamer, AP-9R significantly inhibited the sphere-formation ability of lung cancer cells and the expression of CSC biomarkers. *In vivo*, tumorigenic assays in NOD/SCID mice demonstrated that AP-9R could dramatically suppress tumor proliferation. By blocking Annexin A2-related signaling pathways, such as cytokinesis, endocytosis, exocytosis, and signal transduction, AP-9R reduced cancer stemness in lung cancer [44]. In addition to direct blocking of CSC-related signaling pathways, aptamers can be developed to reverse multidrug resistance (MDR). For example, Ma et al. screened a DNA aptamer targeting ABCG2, a CSC biomarker that plays a key role in multidrug resistance. By simultaneously binding the two monomers of ABCG2 dimers and thereby blocking the ABCG2-mediated drug-pumping channel, the selected aptamer successfully increased the intracellular accumulation of substrate drugs. The MDR reversing capability was described by the reversal fold (RF), which is the ratio of the IC_50_ value of drug to cancer cells in the presence of the aptamer to that in the absence of aptamer. *In vitro* studies indicated that the RF values in this study were 1.62, 1.62, and 1.55, respectively, after incubation with the drug for 24, 36, and 72 ​h. Although the MDR reversing capability was not very significant, this study presented a new way to develop therapeutics for CSC elimination and MDR reversal. Further studies are necessary to explore how to improve the RF values and evaluate the therapy efficacy of ABCG2 aptamers in an animal model [45].

## 3 Conventional ApDCs

Conventional ApDCs are mainly composed of a single-targeting aptamer and a therapeutic agent. They can selectively bind with cancer cells with high expression of the biomarker recognized by the aptamer and kill the targeted cells with the help of the therapeutic cargo [46,47]. To eliminate CSCs in different types of tumors, new conventional ApDCs have been developed in recent years (Table 1).

**Table 1:** Examples of conventional aptamer-based targeted therapies for CSCs.

Molecular targets	Cancer types	Cargoes	Nanocarriers	Modalities	Ref
EpCAM	Hepatocellular carcinoma	DOX	None	Chemotherapy	[29]
CD133	Lung cancer	Gefitinib	DSPE-PEG2000	Chemotherapy	[54]
CD133	Glioblastoma	Telaglenastat	PEGylated gold nanoparticles	Chemotherapy	[106]
CD20	Melanoma	Salinomycin	Lipid-polymer	Chemotherapy	[107]
CD20	Melanoma	Adriamycin	Exosomes	Chemotherapy	[108]
EpCAM	Colorectal cancer	DOX	None	Chemotherapy	[109]
CD133	Thyroid cancer	DOX	None	Chemotherapy	[110]
EGFR	Osteosarcoma	Salinomycin	polymer-lipid hybrid nanoparticles	Chemotherapy	[111]
EpCAM	Hepatocellular carcinoma	β-catenin siRNA	Milk-derived nanovesicles	Gene therapy	[59]
EpCAM	Colorectal cancer	Survivin siRNA	None	Gene therapy	[60]
CTLA4	Chronic myeloid leukemia	Kindlin-3 siRNA	None	Gene therapy	[61]
CD133	Breast cancer	Anti-miR21	three-way junction (3WJ) motif	Gene therapy	[62]
Axl and PDGFRβ	Glioblastoma	miR-137 and antimiR-10 ​b	None	Gene therapy	[63]
EpCAM	Hepatocellular carcinoma	Ad5-PTEN	polyethylene glycol	Gene therapy	[112]
EpCAM	Breast cancer	Upf2, Parp1, Cd47, and Mcl1 siRNA	None	Immunotherapy	[70]

### 3.1 Conventional CSC-targeted ApDCs for chemotherapy

The development of chemotherapy and the discovery of new drugs have dramatically improved the prognosis of cancer [48]. However, conventional chemotherapies cannot specifically target cancer cells and might cause severe damage to healthy tissues and organs [49]. Recently, the unmet needs of cancer treatment have greatly motivated the development of drug delivery systems [50]. As mentioned above, CSC-induced chemotherapy resistance and tumor relapse are the two factors that increase the risk of therapeutic failure. To reduce the side effects of conventional chemotherapies and improve their therapeutic efficacy, some researchers have used aptamers as targeting ligands to guide drugs to CSCs. For high drug delivery efficacy, aptamers are usually combined with different kinds of nanocarriers, including organic nanomaterials and inorganic nanomaterials (Fig. 1B) [51,52].

Zahiri et al. developed CD133-PCAD-DMSN@DOX through a multi-step synthesis process to transport drugs to the site of action. *In vitro* studies demonstrated that CD133-PCAD-DMSN@DOX selectively targeted and successfully enhanced the cytotoxicity of Doxorubicin (DOX) toward CD133-positive cell lines. In the synthesis process, dendrimer-like silica was used as a drug carrier to enhance the efficiency of drug delivery because of its unusual characteristics, such as a special center-radial mesopore structure and widespread pore size. To improve biocompatibility, dextran polycarboxylic acid (PCAD) was attached to the surface of dendrimer-like silica to fully cover the silica surface. Under an acid environment, the PDAC coat breaks, leading to DOX release. Furthermore, CSC targeting was achieved by attaching CD133 aptamers to the surface of the dendritic silica nanoparticles. The apoptosis-inducing effect of CD133-PCAD-DMSN@DOX was investigated using flow cytometry, which demonstrated that Apt-PCAD-DMSN@DOX led to a higher reduction in the population of CD133-positive cancer cells (48%) than the nontargeted formulation (36%) [53]. Similarly, Huang et al. fabricated Poly (ethylene glycol) 2000-distearoylphosphatidylethanolamine (DSPE-PEG2000)-based nanoparticles conjugated with CD133 aptamers to efficiently deliver gefitinib, a tyrosine kinase inhibitor of the epidermal growth factor receptor (EGFR), to target CSCs. *In vitro*, the effect of the constructed nanomicelles (M-Gef-CD133) on CSCs was assessed using tumorsphere formation assays, which showed that M-Gef-CD133 treatment significantly decreased the number of tumorspheres compared with gefitinib or M-Gef alone [54]. Unfortunately, the two studies mentioned above lacked evidence of *in vivo* efficacy.

Meanwhile, their ease of modification and programmability make the aptamer-based drug delivery systems versatile. Drugs can physically or site-specifically chemically conjugate with the folded aptamer, without the help of other nanocarriers. For example, by simply adding folded aptamers to a fixed concentration of DOX in conjugation buffer and incubating the mixture for 2 ​h, Zhou et al. formulated EpCAM-apt-Dox. The CSC-killing effect of EpCAM-apt-Dox *in vitro* was demonstrated via colony-formation assays, which indicated that EpCAM-apt-Dox led to more than 40% reduction in the relative stem cell frequency compared with that of free Dox. *In vivo*, relative to that of the Dox alone treatment group, EpCAM-apt-Dox induced more than 40% reduction in tumor weight and size [29]. In a similar manner, CD133-apt-DOX was constructed to inhibit the self-renewal ability of liver CSCs and attenuate their stemness phenotypes [31].

### 3.2 Conventional CSC-targeted ApDCs for gene therapy

Correcting the genetic error is another promising approach to treat cancer. Gene therapy emerged in recent years and displayed great potential to treat cancers, especially those that are resistant to conventional therapy [55,56]. RNA interference (RNAi) is often used in gene therapy because it can efficiently regulate gene expression. Among several types of RNAi agents, gene silencing using siRNA has become a hot research topic [57]. However, naked siRNA degrades quickly in blood circulation, resulting in low bioavailability. Furthermore, similar to conventional chemotherapies, siRNAs lack the ability to selectively target cancer cells; therefore, it is important to find a carrier to deliver siRNAs specifically to tumor tissues [58]. Towards this end, aptamer-siRNA conjugates have been developed to deliver siRNAs into CSCs (Fig. 1C). For example, based on EpCAM aptamer, Ishiguro et al. designed a therapeutic nanoparticle to deliver siRNAs that could regulate the expression of β-catenin to liver CSCs. To improve the delivery efficiency, milk-derived nanovesicles were coupled with the EpCAM aptamer. *In vitro*, the uptake of EpCAM-targeting therapeutic milk-derived nanoparticles (ET-tMNVs) by CSCs led to knockdown of β-catenin and cell apoptosis. *In vivo*, compared with that in the ET-scramble siRNA-loaded MNV treated mice, the tumor volume was significantly smaller in mice receiving ET-tMNVs [59]. Besides directly regulating the expression of tumor-related genes to inhibit tumor growth, aptamer-siRNA conjugates have also been used to reverse drug resistance. In an independent study, EpCAM aptamer-guided siRNA successfully downregulated the expression of survivin, one of the key factors responsible for the chemoresistance of colorectal CSCs. *In vitro*, tumorsphere formation assays demonstrated that a combination of survivin knockdown and 5-fluorouracil (5-FU) treatment led to a 3-fold decrease in self-renewal, while there was no significant decrease observed in the other groups. *In vivo*, they collected the tumors after treatment with the aptamer-siRNA chimera and 5-FU. Then, the tissues were chopped and dissociated into single cell suspensions. A limiting dilution assay (LDA) was conducted to assess the tumorsphere formation ability, which indicated that the combination of the aptamer-siRNA chimera and 5-FU greatly reduced the self-renewal capacity of colorectal cancer [60]. Significantly, ApDCs that can target and eliminate non-solid tumor chronic myeloid leukemia (CML) CSCs have been developed by Krenn et al. They used an aptamer against cytotoxic T-lymphocyte associated protein 4 (CTLA4) which is highly expressed on leukemic stem cells (LCSs), but not on normal hematopoietic stem cells, to deliver a Kindlin-3 (K3) siRNA to LCSs. *In vivo*, depletion of K3 efficiently decreased the number of LCSs in the bone marrow, prevented LCS dissemination into extramedullary organs, and resulted in long-lasting remission in mice suffering from CML regrowth [61].

In addition to siRNAs, other types of RNAi agents such as microRNAs (miRNAs) and anti-miRNAs, can also regulate the expression of genes of interest. For example, Yin et al. utilized a thermodynamically and chemically stable three-way junction motif as the scaffold and CD133 aptamers as the targeting ligand to deliver a locked nuclei acid sequence, anti-miR21, that binds to intracellular miRNA21 in breast CSCs. *In vitro* and *in vivo* experiments indicated that the nanoparticles could specifically target triple-negative breast cancer and reduce cancer cell migration [62]. In another example, GL21.T and Gint4.T aptamers were used as carriers for miR-137 and antimiR-10 ​b (an miR-10 ​b inhibitor), respectively, to eradicate glioblastoma CSCs. miR-137 is an oncosuppressor in glioblastomas that inhibits cancer cell proliferation and invasion. Meanwhile, miR-10b is overexpressed in glioblastoma, acting as an oncomiR. Unfortunately, this study was solely focused on *in vitro* experiments. Tumorsphere formation assays demonstrated that the combination of GL21.T-miR137 and Gint4.T-anti10b conjugates significantly inhibited the propagation of glioblastoma CSCs and the formation of tumor spheroids [63].

### 3.3 Conventional CSC-targeted ApDCs for immunotherapy

Immunotherapy to treat cancer has achieved impressive success and made significant breakthroughs in recent years [64,65]. While these new therapeutic strategies can significantly promote antitumor immunity [66], most of them lead to autoimmune toxicity; therefore, it is necessary to deliver immunotherapies selectively into tumor cells [67]. Meanwhile, increasing evidence indicates that CSCs interact with infiltrating immune cells with protumoral effects in the tumor microenvironment. For example, tumor associated macrophages (TAMs) can be classified into M1 and M2 phenotypes. M1 macrophages have an anti-tumor effect, while M2 macrophages promote tumor invasion, metastasis, and immune evasion. Notably, infiltration of TAMs is usually related to cancer proliferation [68]. Recent studies indicated that the levels of protumorigenic macrophage factors, such as C–C motif chemokine 2 (CCL2), macrophage colony-stimulating factor 1 (CSF1), and transforming growth factor-β (TGFβ), were elevated in CSCs compared with non-CSCs. CSCs could be essential for monocyte recruitment in various tumor types. Mutually, tumor TAMs promote CSC maintenance via soluble mediators, including interleukin-6 (IL-6) and pleiotrophin (Fig. 1D) [14]. Moreover, another study indicated that a high-stemness signature was related to a poor immunogenic response in 21 types of cancer [69]. Therapeutic strategies that can inhibit the CSC phenotype and simultaneously reduce immunosuppression might display an unexpected synergistic effect. Therefore, some researchers utilized aptamers against CSCs to guide immunotherapeutic agents to treat cancer.

siRNAs targeting a series of immune-related genes, including *Upf2*, *Parp1*, *Apex1*, *Cd47*, *Cd274*, and *Mcl1*, were linked to EpCAM aptamers to selectively knockdown genes in breast CSCs with the aim of overcoming immune evasion. Inhibiting the expression of *Upf2*, *Parp*1, and *Apex1* promoted tumor neoantigen expression. *Cd47* knockdown induced phagocytosis and antigen presentation, and knockdown of *Cd274* and *Mcl1* reduced checkpoint inhibition and caused tumor cell death, respectively. The six immune-related genes were downregulated using EpCAM aptamer-linked siRNA chimeras (EpCAM-AsiCs). The function of EpCAM-AsiCs in the process of cancer immunity was evaluated in genetically engineered mouse breast cancer models, which indicated that *Upf2*, *Parp1*, *Cd4*7, and *Mcl1* knockdown by EpCAM-AsiCs obviously inhibited tumor progression and promoted tumor-infiltrating immune cell functions. Moreover, AsiC mixtures can be easily created from the assembly of individual AsiCs, and the combination of the four most effective EpCAM-AsiCs (targeting *Upf2*, *Parp*1, *Cd47*, and *Mcl*1) exerted a better tumor inhibition effect than the individual EpCAM-AsiCs. Combining EpCAM-AsiCs against multiple pathways could potentially boost immunity toward tumors that are barely responsible for checkpoint blockade [70]. Unfortunately, although the authors pointed out that EpCAM is overexpressed in breast cancer CSCs, they did not perform experiments, such as tumorsphere formation assays, to evaluate the inhibitory effects of EpCAM-AsiCs on CSCs. The discovery of CSC-immune cell crosstalk could pave the way to improve the prognosis of patients with cancer. We anticipate the generation of targeting therapeutic strategies for the simultaneous regulation of CSCs and the immune response.

## 4 Multifunctional CSC-targeted aptamer-based therapeutics

By conjugating multiple components, such as different kinds of tumor-targeting ligands, anticancer drugs, or siRNAs, to a single construct, not only can these multifunctional nanosystems target more cancer cells, but also they can achieve effective synergistic therapeutic treatment [71,72]. Investigations of multifunctional therapeutic strategies are progressing rapidly and provide new opportunities in the treatment of cancer [73,74]. Based on aptamers, researchers have designed multifunctional CSC-targeted drug delivery systems with different modalities in recent years (Table 2).

**Table 2:** Examples of multifunctional aptamer-based targeted therapies for CSCs.

Molecular targets	Cancer types	Cargoes	Nanocarriers	Modalities	Ref
Nucleolin and EpCAM	Breast cancer	PEGylated silver nanotriangles	None	Photothermal therapy	[38]
CD44	Breast cancer	AKTin and DOX	DNA building blocks	Combined chemotherapy	[30]
CD133	Glioblastoma	PTX and TMZ	Polyamidoamine (PAMAM) G4C12 dendrimer	Combined chemotherapy	[89]
CD133	Glioblastoma	TMZ and RG7388	Polymer-micellar NPs	Combined chemotherapy	[90]
CD44 and PD-L1	Breast cancer	DOX and IDO1 siRNA	Nano-liposome	Chemoimmunotherapy	[113]
EGFR and CD133	Osteosarcoma	Salinomycin	Lipid-polymer	Chemotherapy	[78]
MUC1 and CD44	Breast cancer	DOX	Liposomes	Chemotherapy	[36]
LRP and CD133	Glioblastoma	PTX and survivin siRNA	Liposomes	Chemotherapy and gene therapy	[37]
EpCAM and VEGF	Colorectal cancer	MiR-328	Mesoporous silica nanoparticles	Gene therapy	[114]

### 4.1 Multifunctional CSC-targeted ApDCs for co-targeting CSCs and non-CSCs

**Fig. 2:**
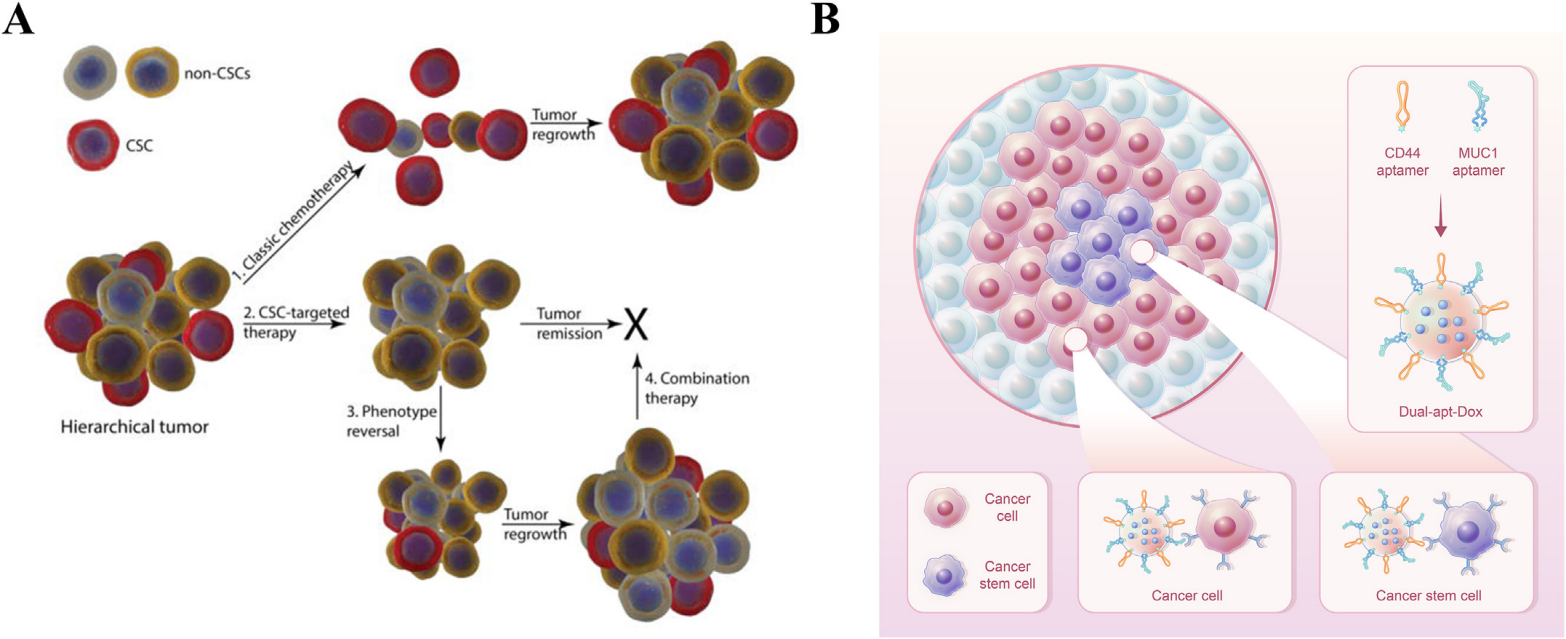
Combination treatments with CSC-targeted and non–CSC–targeted chemotherapy. (A) Treatment of hierarchical tumors. Treatment with classic chemotherapy can reduce the tumor volume; however, CSCs can survive, resulting in tumor regrowth. Therapeutic agents that target and kill CSCs will inhibit the growth of tumors. However, phenotype reversal will result in tumor regrowth. Combination treatment with conventional chemotherapies and drugs targeting CSCs might be an ideal regimen to obtain satisfactory results. Reprinted with the permission from Ref. [10]. **(B)** An example of the application of dual-ApDCs to deliver drugs to both cancer cells and CSCs.

The central tenet of targeted therapy depends on the binding affinity of targeting ligands toward cancer cells [75,76]. However, because of the heterogeneity of biomarkers in different cancer cell subtypes, a single targeting ligand is unable to recognize all cancer cells. Therefore, dual-ApDCs that can simultaneously identify and interact with two different biomarkers were developed for more precise targeting [77]. Meanwhile, classical chemotherapies can hardly kill CSCs and might result in tumor regrowth, which has greatly encouraged the development of CSC-targeted therapeutics. Moreover, according to the dynamic CSC model, non-CSCs have the ability to reverse their phenotype and obtain CSCs properties, which can also lead to tumor relapse (Fig. 2A). Therapeutics that simultaneously eliminate both CSCs and non-CSCs might achieve a more satisfactory response [10].

Kim et al. developed anti-MUC1/CD44 dual-ApDCs to co-target the surface biomarker mucin 1 (MUC1) on breast cancer cells and CD44 on breast CSCs. *In vitro*, dual-aptamer-conjugated liposomes harboring DOX (dual-Apt-DOX) exerted a higher binding affinity and better anticancer effect on both cancer cells and CSCs than DOX delivered by a single aptamer. To evaluate the inhibitory impact of dual-Apt-DOX on the metastasis of breast CSCs and cancer cells *in vivo*, equal numbers of breast cancer cells and CSCs were injected into nude mice via the tail vein, which were subsequently injected with dual-Apt-Dox in the same manner. After 5 weeks, the mice were sacrificed and the tumor colonies in the lungs were observed. The results showed that dual-Apt-Dox displayed a significant inhibitory effect on the metastatic progression of breast cancer compared with that of the control groups (Fig. 2B) [36]. Similarly, Chen et al. used two aptamer-based nanoparticles to deliver salinomycin (sali) to both osteosarcoma cells and CSCs. They constructed sali-entrapped lipid-polymer nanoparticles labeled with CD133 and EGFR aptamers (CESP) and tested its efficacy to inhibit tumor growth through a series of *in vitro* and *in vivo* experiments. The results demonstrated that CESP could efficiently co-target both osteosarcoma cells and CSCs, and their anti-tumor effect was superior to that of the single aptamer-loaded nanoparticles [78].

### 4.2 The combination of aptamers and other kinds of targeting ligands

Besides dual-aptamer-based drug delivery systems, combinations of a single aptamer and other kinds of targeting ligands can also improve the efficacy of drug delivery. In recent years, imaginative multifunctional aptamer-based therapeutic strategies that effectively inhibit CSCs have been developed.

**Fig. 3:**
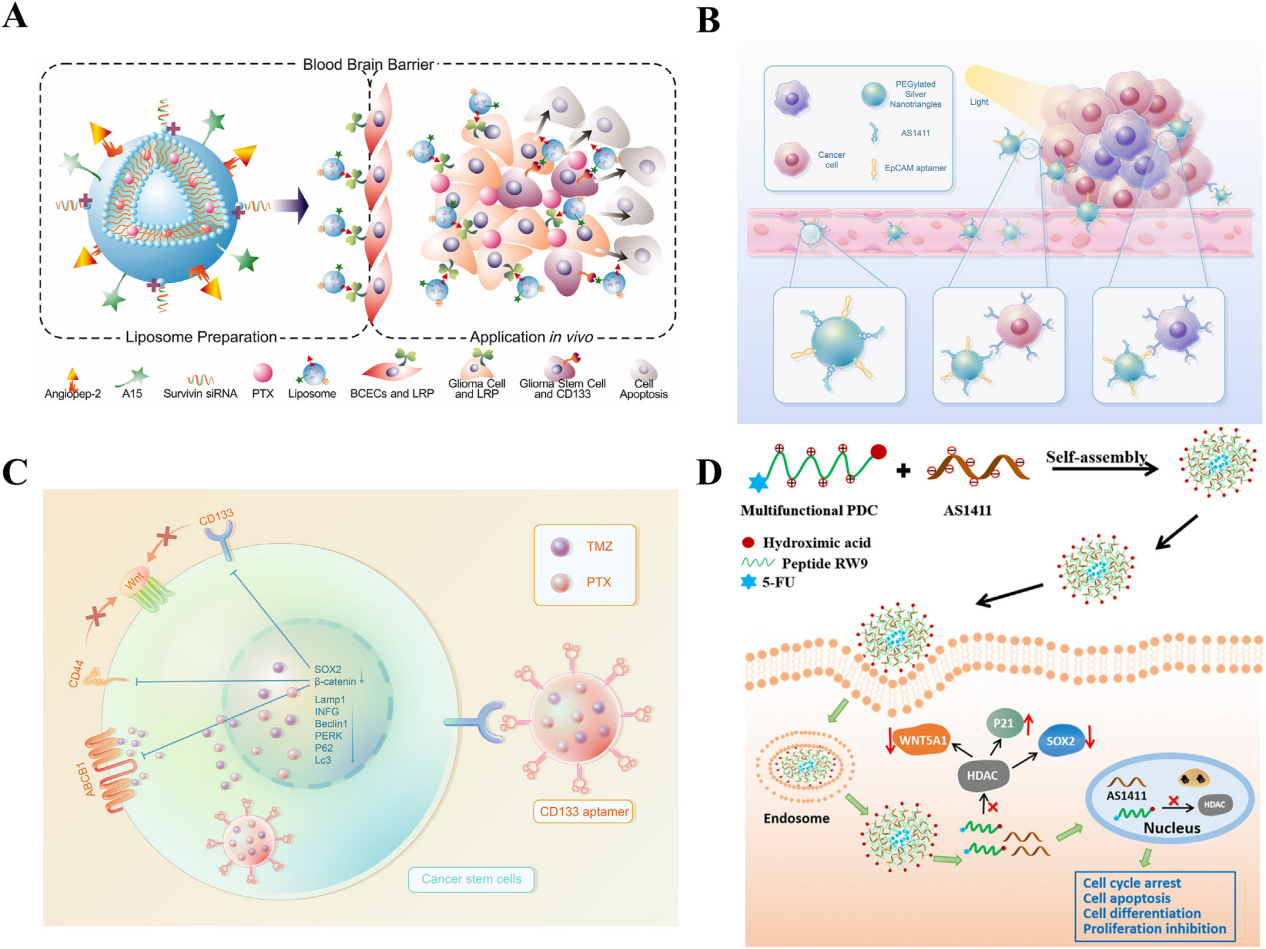
Different modalities of multifunctional aptamers based CSC-targeted therapeutic strategies. (A) Multifunctional aptamer-based CSC-targeted therapeutic strategies containing a single aptamer and other kinds of targeting ligands can simultaneously bind to CSCs and non-CSCs. Reprinted with the permission from Ref. [37]. **(B)** The use of aptamers as targeting ligands to co-target non-CSCs and CSCs for photothermal therapy. **(C)** An example of the application of ApDCs to deliver two drugs to CSCs. In response to Apt-NPs, the SOX2-CD133, CD133-Wnt/β-catenin, and CD44-Wnt/β-catenin axes were downregulated and the expression of autophagy genes, such as *LAMP1*, *INFG*, and *BECN1*, were inhibited. Drug-loaded Apt-NPs could reverse drug resistance in GSCs via downregulation of the Wnt/β-catenin pathway and autophagy genes. **(D)** Multifunctional aptamer-based therapeutics target CSC pathways and thereby inhibit the proliferation of tumors. Reprinted with the permission from Ref. [95].

The blood-brain barrier (BBB) and the blood-tumor barrier (BTB) are natural barriers to the treatment of brain glioma because they hinder the uptake and accumulation of therapeutic agents by tumors. The special anatomical location of glioma means that specifically delivering drugs to glioma stem cells (GSCs) is more challenging than to other cancer types. To address these problems, Sun et al. designed a novel dual-targeting ligand by fusing angiopep-2, which can enhance drug delivery across BTB and BBB to glioma cells by targeting low-density lipoprotein receptor-related protein (LRP), and CD133 aptamer into one unit. To enhance drug delivery efficiency, lipid components were modified with the dual-targeting ligand. Finally, the resulting dual-modified cationic liposomes (DP-CLPs) were used to selectively deliver a combination of paclitaxel (PTX) and a survivin siRNA to glioma cells and especially GSCs. *In vitro*, PTX alone or nonmodified CLPs–PTX–survivin siRNA demonstrated negligible inhibition of CSC growth; however, DP-CLPs–PTX–survivin siRNA treatment exhibited a prominent inhibitory effect on CSC growth. *In vivo*, the fluorescence signal in tumor-bearing brains treated with DP-CLPs–PTX–survivin siRNA was stronger than that of nonmodified CLPs–PTX–survivin siRNA, and the tumor size in the DP-CLPs–PTX–survivin siRNA-treated group was significantly reduced compared with that in the control groups, including PTX alone and nonmodified CLPs–PTX–survivin siRNA (Fig. 3A) [37]. In another study, Wang et al. developed an AS1411 aptamer/hyaluronic acid-bifunctionalized microemulsion for the codelivery of shikonin and docetaxel (AS1411/SKN&DTX-M). AS1411/SKN&DTX-M consists of two targeting ligands (the AS1411 aptamer and hyaluronic acid) and two drugs (shikonin and docetaxel). The AS1411 aptamer and hyaluronic acid can respectively recognize nucleolin and CD44. *In vitro*, AS1411/SKN&DTX-M downregulated the expression of CD133 and induced a 16.9-fold decrease in the area of CSC spheres compared with that in the nontreated group. *In vivo* studies demonstrated that AS1411/SKN&DTX-M could not only significantly inhibit the growth of glioma, but also improved the brain-specific accumulation of therapeutic agents [79]. According to practical needs, different combinations of aptamers and other kinds of targeting ligands could be developed in the future.

### 4.3 Multifunctional CSC-targeted aptamer-based targeted therapies for photothermal therapy

PTT is a newly developed treatment strategy, aiming to improve the therapeutic outcome of cancer [80,81]. In PTT, a photothermal effect is achieved through the use of photothermal transduction agents (PTAs). Under the irradiation of light at a specific wavelength, PTAs harvest energy from photons and convert the energy into heat, consequently increasing the temperature of the surrounding microenvironment and leading to the death of cancer cells [82,83]. To enhance the outcome and reduce the side effects, it is necessary to increase the accumulation of PTAs in tumor tissues. Targeting strategies that can guide PTAs to their sites of action have received increased research attention [84,85]. Recently, aptamers have been explored as targeting ligands for the active delivery of PTAs.

Silver nanotriangles proved to be effective PTAs in PTT [86]. To deliver them specifically to breast cancer cells and breast CSCs, the AS1411 and EpCAM aptamer were conjugated to PEGylated silver nanotriangles. *In vitro*, breast CSCs were enriched from serum free medium, and the effect of the resulting AS1411 and EpCAM aptamer-conjugated PEGylated silver nanotriangles (AENTs) on breast CSCs was evaluated using the MTT assay. The results showed AENTs had the greatest inhibitory effect among all the groups. Wound healing and Transwell invasion assays were performed to assess the effects of AENT-mediated PTT on the migration and invasion of breast cancer cells. When combined with an 808 ​nm near-infrared (NIR) laser, AENTs could significantly inhibit cell migration and invasion, with the greatest inhibitory effects (48.2%) and the lowest invasion rate (22.8%). *In vivo*, after being injected with the nanomaterials and irradiated with the NIR laser, the tumor temperature in mice was measured, and the results indicated that the temperatures of AS1411-conjugated PNTs (ANTs) and AENT groups were about 55 ​°C, which was higher than those of the other groups. At 20 days after treatment, the mice were sacrificed, and the results showed that AENTs plus NIR displayed the strongest anticancer effect among all the treatments (Fig. 3B) [38]. Killing CSCs using PTT is a significant breakthrough. It is expected that more new therapeutic strategies will be conjugated to aptamers to target and eliminate CSCs.

### 4.4 Multifunctional CSC-targeted ApDCs for the co-delivery of two therapeutic agents

Chemotherapy, one of the main therapeutic strategies in cancer treatment, is used clinically for both palliative and curative purposes. However, because of tumor heterogeneity and gene mutation, many cases of cancer show a poor response to assigned chemotherapies. The occurrence of MDR greatly limits the application of certain drugs. Compared with a single therapeutic modality, co-administration of two kinds of therapeutic agents can reduce the dose of single drug, lower the toxicities in patients, and decrease drug resistance and the adverse effect of a monotherapy agent [87,88]. To achieve the optimal tumor inhibition effect, CSC-targeted ApDCs that co-deliver two agents have been developed.

For example, to reduce tumor recurrence and chemotherapy resistance of glioblastoma multiforme, CD133 aptamer-conjugated polyamidoamine G4C12 dendrimer nanoparticles (Apt-NPs) were developed to co-deliver temozolomide (TMZ) and PTX to GSCs. *In vitro*, anti-proliferation assays showed that the drug-loaded Apt-NPs greatly inhibited the growth of CSCs. In addition, because of the different mechanisms of the two drugs and their ability to specifically target CSCs, the drug-loaded Apt-NPs exerted a synergistic effect and resulted in a better anticancer effect compared with treatment using either single drug or with the combination of PTX and TMZ. However, this study was not perfect, and *in vivo* experiments are necessary to further clarify the results (Fig. 3C) [89]. In another study, to enhance the efficacy of TMZ, polymer-micellar nanoparticles were developed for the co-delivery of TMZ and RG7388 (an inhibitor of glioblastoma multiforme DNA damage response systems) and covalently bound to the CD133 aptamer to target CSCs. When TMZ and RG7388 were utilized in combination, dual drug-loaded nanoparticles exhibited a much higher killing effect on CSCs than using TMZ-loaded nanoparticles alone. Unfortunately, this study also lacked *in vivo* support. Additional studies are warranted to evaluate the therapeutic efficacy of the NPs in a preclinical model of GBM [90]. Similarly, Xu et al. formulated an aptamer-conjugated DNA nanotrain, TA6NT-AKTin-DOX, which comprised a CD44 aptamer, DOX, and DNA building blocks M1 and M2 conjugated with peptide AKTin (an inhibitor of protein kinase B (AKT)) individually, to target and eliminate breast CSCs. *In vitro*, TA6NT-AKTin-DOX exhibited a significant inhibitory effect on the formation of tumorspheres, with the number of spheres being reduced by 57.1 ​± ​1.5% relative to the nontreated group. *In vivo*, in mice treated with TA6NT-AKTin-DOX, their tumor weight was much lower than that of mice treated with free DOX [30]. The designed multidrug-loaded nanoparticles guided by an aptamer against CSC biomarkers represent a new therapeutic strategy to maximize drug efficacy and reverse drug resistance.

### 4.5 Multifunctional aptamer-based therapeutics to target CSCs pathways

In addition to serving as a targeting ligand by interacting nucleolin, aptamer AS1411 can kill cancer cells through different molecular mechanisms [91,92]. To efficiently deliver AS1411 to the nucleus and achieve a synergistic antitumor effect, Wang et al. designed a multifunctional peptide drug conjugate (PDC) comprising the cell penetration peptide RW9, 5-FU at the peptide's N-terminus, and an HDAC inhibitor (HDACi) warhead at the peptide's C-terminus. Many studies have suggested that HDAC is associated with cell differentiation and the expression of genes related to the cell cycle in CSCs [93,94]. For example, HDAC inhibition could lead to the downregulation of the stemness gene SRY-box transcription factor 2 (SOX2), which is highly correlated with CSC differentiation. In the study by Wang et al., the peptide was used as nucleic acid delivery vehicle, AS1411 was considered as nucleic acid drug, and HDACi and 5-FU were attached to the nanoparticle to exert a synergistic antitumor effect. Although the authors only conducted *in vitro* experiments, the results demonstrated that the combination of HDACi, 5-FU, and AS1411 showed selective toxicity to CSCs, but not to normal 293 ​T cells, and inhibited cancer proliferation through multiple signaling pathways, such as cell cycle arrest, inducing cell apoptosis, downregulation of stemness protein SOX2 and cancer-related protein WNT5A1. In fact, AS1411 served as neither a targeting ligand against CSCs nor a blocker for CSC-related pathway in this study; however, the results demonstrated that the PDC showed unexpected synergy with AS1411 to augment the efficiency of CSC and non-CSC suppression (Fig. 3D). This study presented a new modality of aptamer-based therapeutics against CSCs [95].

## 5 Cell-SELEX

**Fig. 4:**
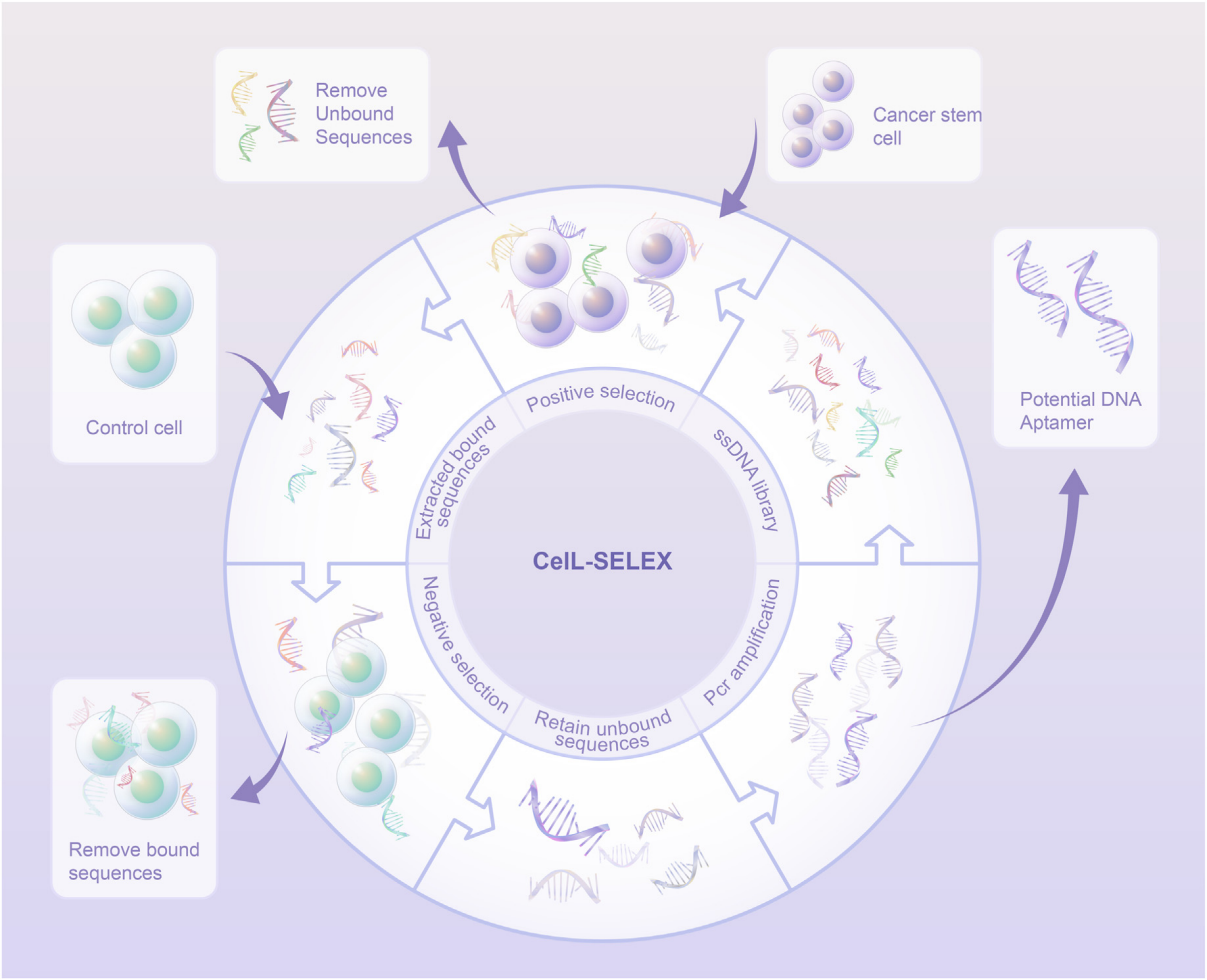
Schematic representation of selecting DNA aptamers against CSCs using cell-SELEX. A single-stranded DNA (ssDNA) library pool is incubated with the CSCs. Non-binding sequences are removed and bound sequences are recovered from the cells. The recovered pool is incubated with the control cells to filter out the sequences that bind to common molecules.

Aptamers against CD44, CD133, and EpCAM have been tested as targeting ligands in a variety of tumor types. As indicated in Table 3, the most common CD133 aptamer used for research has the sequence: 5′-CCCUCCUACAUAGGG-3′, and EpCAM aptamer with the sequences 5′-ACGUAUCCCUUUUCGCGU-3′ or 5′-GCGACUGGUUACCCGGUCG-3′ is frequently used as a targeting ligand for CSCs in different tumor types. Some researchers took advantage of the existing aptamers to design aptamer-based targeted therapy for CSCs, while the others identified new aptamers that might have a higher affinity for CSCs. Generally, SELEX is an efficient technology to generate aptamers [96,97]. In the SELEX process, the incubated target molecules are usually proteins or peptides; however, the whole cell can also be the target [98]. In the case of cell-SELEX, an oligonucleotide library is incubated with whole cells to generate highly specific aptamers (Fig. 4) [99]. Furthermore, even if there is no knowledge of the molecular signatures, we can still generate aptamers for the cells of interest through cell-SELEX. Thus, researchers have screened some new aptamers to selectively target CSCs using this technology.

**Table 3:** Sequences of aptamers against CSCs.

Aptamer	Sequence	Ref
EpCAM	5′-ACGUAUCCCUUUUCGCGU-3′	[29]
EpCAM	5′-GCGACUGGUUACCCGGUCG-3′	[38]
EpCAM	5′-GCGACUGGUUACCCGGUCG-3′	[59]
EpCAM	5′-ACGUAUCCCUUUUCGCGU-3′	[60]
EpCAM	5′-GCGACUGGUUACCCGGUCG-3′	[70]
EpCAM	5′-ACGUAUCCCUUUUCGCGU-3′	[109]
EpCAM	5′-GCGACUGGUUACCCGGUCG-3′	[112]
EpCAM	5′-CACTACAGAGGTTGCGTCTGTCCCACGTTGTCATGGGGG GTTGGC CTG-3′	[114]
CD133	5′-CCCUCCUACAUAGGG-3′	[37]
CD133	5′-CCCUCCUACAUAGGG-3′	[54]
CD133	5′-GUCUUAUCAUAUGCAAGAC-3′	[62]
CD133	5′-GCCUUAGUAACGUGCUUUGAUGUCGAUUCGACAGGA GGC-3′	[78]
CD133	5′-CAGAACGUAUACUAUUCUG-3′	[89]
CD133	5′-CCC UCC UAC AUA GGG-3′	[90]
CD133	5′-CCCUCCUACAUAGGG-3′	[106]
CD133	5′-TACCAGTGCCGTTTCCCCGGAGGGTCACCCCTGACGCAT TCGGTTGAC-3′	[110]
CD44	5′-GAGATTCATCACGCGCATAGTCTTGGGACGGTGTTAAAC	[30]
	GAAAGGGGACGACCGACTATGCGATGATGTCTTC-3’	
CD44	5′-CCAAGGCCTGCAAGGGAACCAAGGACACAGTTTTTTTT TT -3′	[36]
CD44	5′-CCAAGGCCTGCAAGGGAACCAAGGACACAGTTTTTTTT TT -3′	[113]
CTLA4	5′-GGGAGAGAGGAAGAGGGAUGGGCCGACGUGCCGCA-3′	[61]
Axl	5′-AUGAUCAAUC GCCUCAAUUCGACAGGAGGCUCAC-3′	[63]
PDGFRβ	5′-UGUCGUGGGGCAUCGAGUAAAUGCAAUUCGACA-3′	[63]
CD20	5′-CTCCTCTGACTGTAACCACGCCGTATGTCCGAAATACG GAGAACAGCACTCATATGCAAGCCATACGCGGAGGTGCACGCGCATAGGTAGTCCAGAAGCC-3′	[107]
CD20	5′-CTCCTCTGACTGTAACCACGCCGTATGTCCGAAATACG GAGAACAGCACTCATATGCAAGCCATACGCGGAGGTGCAC GCGCATAGGTAGTCCAGAAGCC-3′	[108]
EGFR	5′-GCCUUAGUAACGUGCUUUGAUGUCGAUUCGACAGGA GGC-3′	[111]

For instance, Wu et al. used glioma CSCs as target cells and U87 ​cells as counter selection negative cells for cell-SELEX. After 20 rounds of selection, five aptamers, W1, W2, W5, W6, and W33, were selected. However, large aptamers have less application value *in vivo* because they have low permeability in tumor tissue and a high synthesis cost. To select a shorter aptamer with better properties, a series of experiments were carried out, and the shortened aptamer W5-7 was verified to be the most applicable aptamer to target CSCs [32]. In another study, to find an aptamer with high specificity for breast CSCs, Lu et al. used cell-SELEX to isolate the aptamer from single-stranded DNA (ssDNA) pools. After 13 rounds of selection, aptamer MS03 was selected because of its high binding affinity for breast CSCs [100]. Cell-SELEX is a well-established method to identify novel molecular biomarkers of CSCs, and the screened aptamers are promising probes for diagnostic and therapeutic applications in different tumor types. However, some problems remain be solved. Although these new aptamers have been screened, their anticancer properties when serving as targeting ligands is rarely known. Various therapeutic cargoes should be conjugated to these novel aptamers to verify their ability to guide drugs to CSCs.

## 6 Conclusion

The application of various treatments for cancers in the clinical works has significantly improved patient survival rates. However, chemotherapy resistance and tumor relapse after treatment usually lead to an unsatisfactory curative effect, which can be attributed to the existence of CSCs [101,102]. Therefore, therapeutic strategies that target and eliminate CSCs have received increased research interest [103,104]. Meanwhile, since aptamers were first discovered, the unique properties of aptamers have promoted their development as alternatives to antibodies for various applications, including biomarker discovery and drug delivery [105]. In this review, we systematically discussed the recent advances in aptamer-based targeted therapies for CSCs. As mentioned above, various therapeutic agents have been attached to aptamers to eradicate CSCs. In addition, new aptamers against CSCs are being screened using cell-SELEX to identify more applicable sequences. The development of multifunctional CSC-targeted ApDCs opens up a way to improve drug delivery efficacy and anticancer effects. In preclinical studies, these aptamer-drug conjugates showed great potential to target and eliminate CSCs.

However, there are several obstacles that hinder the development of aptamer-based targeted therapies for CSCs. Further research should focus on the following aspects. Firstly, multifunctional delivery systems are the key trend for malignant tumor treatment, and different modalities of multifunctional CSC-targeted aptamer-based therapeutic strategies should be designed and tested in different tumor types. Secondly, increasing evidence indicates that there is reciprocal communication between CSCs and immune cells during tumor progression; however, the potential of ApDCs to simultaneously attenuate CSC maintenance and reduce immune evasion remains unknown. Thirdly, current studies usually focus on several aptamers including CD44, CD133, and EpCAM or several cancer types, such as glioma and breast cancer. Additional studies should be devoted to finding more suitable aptamers that target CSCs in different types of cancer. Lastly, some studies lack *in vivo* evidence; thus, it is unknown whether these designed nanoparticles can exert their functions *in vivo*. Going forward, future studies should focus on solving the aforementioned problems to promote the clinical application of CSC-targeted aptamer-based therapeutic strategies. Aptamer-based CSC-targeted therapy is a potential approach to break through the limitations of current therapeutic strategies, thus this field deserves more research attention.

## Declaration of competing interest

The authors declare that they have no known competing financial interests or personal relationships that could have appeared to influence the work reported in this paper.

## Data Availability

The data that has been used is confidential.
